# Nitrate enrichment does not affect enteropathogenic *Escherichia coli* in aquatic microcosms but may affect other strains present in aquatic habitats

**DOI:** 10.7717/peerj.13914

**Published:** 2022-09-27

**Authors:** Meredith T. Davis, Adam D. Canning, Anne C. Midwinter, Russell G. Death

**Affiliations:** 1 School of Natural Sciences, Massey University, Palmerston North, Manawatu, New Zealand; 2 Molecular Epidemiology and Veterinary Public Health Laboratory—Hopkirk Research Institute, School of Veterinary Science, Massey University, Palmerston North, Manawatu, New Zealand; 3 Centre for Tropical Water and Aquatic Ecosystem Research (TropWATER), James Cook University of North Queensland, Townsville, Queensland, Australia

**Keywords:** *E. coli*, Enteropathogenic, Eutrophication, Microcosm, Nitrate, Water quality

## Abstract

Eutrophication of the planet’s aquatic systems is increasing at an unprecedented rate. In freshwater systems, nitrate—one of the nutrients responsible for eutrophication—is linked to biodiversity losses and ecosystem degradation. One of the main sources of freshwater nitrate pollution in New Zealand is agriculture. New Zealand’s pastoral farming system relies heavily on the application of chemical fertilisers. These fertilisers in combination with animal urine, also high in nitrogen, result in high rates of nitrogen leaching into adjacent aquatic systems. In addition to nitrogen, livestock waste commonly carries human and animal enteropathogenic bacteria, many of which can survive in freshwater environments. Two strains of enteropathogenic bacteria found in New Zealand cattle, are K99 and Shiga-toxin producing *Escherichia coli* (STEC). To better understand the effects of ambient nitrate concentrations in the water column on environmental enteropathogenic bacteria survival, a microcosm experiment with three nitrate-nitrogen concentrations (0, 1, and 3 mg NO_3_-N /L), two enteropathogenic bacterial strains (STEC O26—human, and K99—animal), and two water types (sterile and containing natural microbiota) was run. Both STEC O26 and K99 reached 500 CFU/10 ml in both water types at all three nitrate concentrations within 24 hours and remained at those levels for the full 91 days of the experiment. Although enteropathogenic strains showed no response to water column nitrate concentrations, the survival of background *Escherichia coli*, imported as part of the in-stream microbiota did, surviving longer in 1 and 3 mg NO_3_-N/Lconcentrations (*P* < 0.001). While further work is needed to fully understand how nitrate enrichment and in-stream microbiota may affect the viability of human and animal pathogens in freshwater systems, it is clear that these two New Zealand strains of STEC O26 and K99 can persist in river water for extended periods alongside some natural microbiota.

## Introduction

The provision of clean water and sanitation for all by 2030 is one of the United Nations Sustainable Development Goals (United Nations, 2015; United Nations, 2020). Although increased access to consistently sanitised water has reduced the number of drinking water related outbreaks in many developed countries, there are currently 1.8 billion people worldwide lacking safely managed drinking water supplies (United Nations, 2020) including portions of New Zealand (Ministry of Health, 2018). In many regions, sources of drinking water are contaminated with faeces and nutrients as a result of current and historical wastewater, agricultural, and industrial practices (Baron et al., 2002; Mallin et al., 2015; Jalliffier-Verne et al., 2016; United Nations, 2016). Source water quality is particularly important in areas where water sanitation measures are absent or prone to failure, where microorganisms are resistant to the sanitation treatments available, and/or where recreational, food irrigating, and/or food harvesting waters are affected (Hrudey, Hrudey & Pollard, 2006; Jones et al., 2013; Viñas, Malm & Pettersson, 2019). To achieve the United Nation’s 2030 goal, preventing and remediating source water pollution in combination with increased water sanitation infrastructure is necessary (United Nations General Assembly, 2015; United Nations, 2020).

Globally, agriculture uses more than 70% of all freshwater abstractions, with livestock farming using disproportionately more water than other agricultural products (*e.g.*, grain and vegetables) by weight (Pimentel et al., 2004). Once used, that water commonly re-enters the environment polluted with both nutrients and faeces (Pimentel et al., 2007; Khan & Mohammad, 2014). In New Zealand, land use has changed considerably over the last few decades, moving away from beef and sheep farming to a more intensive pastoral dairy model (Weeks et al., 2016; Julian et al., 2017). Between 1990 and 2015, New Zealand agriculture documented a 600% increase in fertiliser use, a 90% increase in irrigated agricultural land, and a 70% increase in the number of dairy cattle, with at least 65% of animal-derived nitrate leachate into freshwater ecosystems originating from dairy farms (Stats, 2017). As a result, New Zealand’s freshwaters have amassed considerable levels of faecal and nutrient pollution (Gluckman, 2017; Stats, 2020).

Waterborne disease is a not just a third world problem. The largest drinking water acquired campylobacteriosis outbreak ever documented happened in 2016 in Havelock North, New Zealand (Gilpin et al., 2020). More than 5,500 people using an unchlorinated town water supply developed symptoms of gastroenteritis after the water was likely contaminated with sheep faeces (Gilpin et al., 2020).

In New Zealand, between 2013 and 2018, just 32% of 364 monitored drinking water sites met the *Escherichia coli* (*E*. *coli*) standards at all times (Ministry of Health, 2018). Median nitrate-nitrogen (NO_3_-N) levels at 44% of monitored sites were greater than 3 mg NO_3_-N /L, a standard limit used by Regional Councils for reporting, and 19% of sites failed to meet safe drinking water standards for nitrate with levels greater than 11 mg NO_3_-N /L, at least once (Stats, 2020). Additionally, it is estimated that 50% of New Zealand’s rivers (by length) are above 0.44 mg NO_3_-N/L (ANZG, 2018). A recent report by Environment Canterbury (New Zealand) Regional Council (2020) concluded that the observed deterioration of regional ground water quality in the region was a direct result of agricultural land use. Of the 202 wells tested for NO_3_-N across the region 47% were actively deteriorating and 28% were unchanged (Tregurtha, 2020). Of those, 32% had nitrate levels of greater than 5.65 mg NO_3_-N/L, half of the maximum acceptable value (MAV) (Ministry of Health, 2018), and 6% were above the maximum acceptable value of 11.3 mg NO_3_-N/L. In addition to high NO_3_-N levels, half (102 wells) exceeded the maximum allowable levels of *E*. *coli* between 5% and 50% of the time (Tregurtha, 2020).

Allochthonous human or animal pathogenic organisms in aquatic habitats are a primary cause of disease in humans and animals (Schwarzenbach et al., 2010; Jenkins, Ahmed & Barnes, 2021), commonly associated with faecal contamination and typically assessed by monitoring water column *E*. *coli* levels as a proxy (Odonkor & Ampofo, 2013). One cow/pig/sheep excretes approximately twice the *E*. *coli* per day a human does, some of which may be human and/or animal enteropathogenic strains (Jamieson et al., 2003; Avery, Moore & Hutchison, 2004). Shiga-toxin producing *E*. *coli* (STEC) are one type of zoonotic bacteria commonly hosted asymptomatically by ruminants and shed through their faeces (Cooley et al., 2013). *Escherichia coli* strains, especially strains expressing Shiga-toxins capable of causing diarrhoea, enterohaemorrhagic disease or haemolytic uraemic syndrome in humans are clinically and economically significant emerging zoonoses both in New Zealand and globally (Centers for Disease Control and Prevention, 2018).

Instead of identifying human or animal pathogenic organisms directly in aquatic systems, non-specific *E*. *coli* in the water column are monitored, as their presence is assumed to be representative of the extent of recent faecal contamination (Odonkor & Ampofo, 2013). However, *E*. *coli*, including enteropathogenic strains such as STECs, are able to persist for months or years outside a host (Fairbrother & Nadeau, 2006; Fremaux, Prigent-Combaret & Vernozy-Rozand, 2008; Ahmed, Gyawali & Toze, 2015). Two strains of STEC, responsible for the majority of human disease globally, O157 and O26 are frequently associated with environmental, produce, and/or water contamination (Centers for Disease Control and Prevention, 2018; Joseph et al., 2020).

Other *E*. *coli* strains hosted by ruminants (*e.g.*, enteropathogenic K99) are a leading cause of diarrhoeal disease and mortality in neonatal livestock (Brunauer, Roch & Conrady, 2021).

In addition to *E*. *coli*, other microorganisms (*e.g.*, viruses, protozoa, and other bacteria) responsible for both human and animal disease present in livestock faeces can enter adjacent aquatic systems where environmental stores may be created (Soller et al., 2010; Salman & Steneroden, 2015).

Understanding the mechanistic drivers of enteropathogenic bacteria in aquatic systems is complex. Livestock dominated catchments frequently display heavily modified geography; altered hydrological cycling, significant soil compaction, and/or increased erosion (Germer et al., 2010). Additionally, reduced riparian cover, agrochemical application, and nutrient leaching is common (Germer et al., 2010; Molina et al., 2017). These modifications may affect the movement, virulence, and/or persistence of human and animal pathogens (Strawn et al., 2013). This is concerning, as many livestock hosted microorganisms are potentially zoonotic and shed through excreta, urine and faeces (Cleaveland, Laurenson & Taylor, 2001).

Of these changes, eutrophication is one of the most pervasive, impacting aquatic systems globally (Frumin & Gildeeva, 2014). Nutrient enrichment can affect aquatic microorganisms directly by relieving nutrient-limited growth constraints or indirectly through reduced predator suppression (Haller et al., 2009; Zimmer-Faust et al., 2017). Experimentally manipulating aquatic environmental conditions and assessing the growth and persistence of organisms of interest is a useful way to gain a better understanding of the individual drivers of waterborne bacteria.

Studies of in-stream microbial communities have suggested that predation by, and competition with, in-stream microbiota are the primary factors limiting *E*. *coli* survival in the water column (Wanjugi & Harwood, 2013a; Korajkic et al., 2019b). Despite extensive research into *E*. *coli,* including work showing that non-toxigenic *E. coli* can take up nitrate (Taabodi et al., 2019), the influence of concentrations of nitrate (the most abundant form of dissolved inorganic nitrogen in waterways Sigman et al., 2001) on the growth and persistence of *E*. *coli* in aquatic systems, remains largely unexamined.

This study aimed to use a microcosm experiment to investigate the effects of ambient nitrate concentrations in the water column on survival rates of two enteropathogenic strains of *E*. *coli*, one affecting calves (K99) and one affecting humans (STEC O26), in both a sterile environment and in the presence of in-stream microbiota. We hypothesised there would be a positive relationship between water column nitrate concentrations and pathogenic *E*. *coli* reproduction and persistence. We also anticipated that the presence of in-stream microbiota would mediate this relationship, as microbial predation and/or competition may limit the survival of the pathogenic *E*. *coli* (Wanjugi & Harwood, 2013b). Although *E*. *coli* stores are most commonly found in benthic substrates (Havelaar & Melse, 2003; Muirhead et al., 2004), this experiment was focused on the effects of water column nitrate concentrations as this is the most common sample type used in monitoring drinking and recreational waters (World Health Organization, 2011; Ministry for the Environment, 2020).

## Materials & Methods

### *Escherichia coli* strains

We used *E*. *coli* K99 (ESR 3020 (ESR—NZRM culture collection)) and STEC O26 (NZRM 3537 (Browne et al., 2018; ESR—NZRM culture collection)), as model organisms and monitored the number of colony forming units (CFU) present in the water column, to measure persistence, over 91 days. A pilot experiment demonstrated that there was no measurable difference between the survival rates of two different STEC strains (*e.g.*, STEC O157 and STEC O26 (Supplementary Information)), therefore, a single STEC strain, O26, was chosen for use in the microcosm.

In addition to the artificially inoculated strains (*i.e.,* K99 and STEC O26), background *E*. *coli*, imported as part of the microbiome in the stream water used, was present in all the wells using unsterilised water. The background *E*. *coli* were present in the control wells at each of the three NO_3_-N concentrations and were monitored in the same manner as the inoculated wells to determine whether they responded to NO_3_-N concentration.

### Microcosm experiment

The nitrate concentrations examined (1 and 3 mg NO_3_-N/L) were selected to align with the proposed New Zealand maximum nitrate levels for riverine ecosystem health (*i.e.,* 1 mg NO_3_-N/L) and three times that level (Essential Freshwater Science and Technical Advisory Group, 2019; Canning, 2020).

Throughout the experiment the water temperature was maintained at ∼10 °C, mimicking a typical New Zealand river’s average winter water temperature (Scott & Poynter, 1991). The temperature-controlled room housing the microcosms had standard UV grow lights set on a timer to mimic a typical winter day length in Palmerston North, New Zealand (−40.393560, 175.633072): 9.5 hr of light and 14.5 hr of dark. New Zealand streams experience the greatest rainfall in winter and consequently the greatest loading of faecal pollution (Phiri et al., 2020).

The experiment used 90 microcosm wells (30L ×10D ×20W cm, containing 3L of water) made in-house. Treatments were replicated five times and randomly assigned to wells.

Two types of water were used in the experiment, (1) sterile stream water, unfiltered and containing cellular debris and other native chemicals, and (2) intact stream water, with the water column microbiome intact. A pilot experiment demonstrated that there was no measurable difference between the use of highly filtered Milli-Q water and unfiltered sterile stream water on K99 or STEC O26 growth or persistence (Supplementary Information).

Stream water was collected from the Turitea stream, Palmerston North, New Zealand (40.393728°S, 175.632937°W). The Turitea is a third order, stony bottom stream, with a five year *E*. *coli* median attribution band rating of E (*i.e.,* the lowest/worst ranking). It typifies the worst 25% of New Zealand streams (LAWA, 2020). Because it drains low intensity agriculture it was less likely these strains would be novel to the in-stream microbiota.

Stream water was left intact until NO_3_-N levels measured less than 0.1 mg/L (within ±5%, +0.1 mg/L) on a TriOS NICO nitrate meter (KISTERS AG, Germany) and then was sterilised or used intact. Stream water was autoclaved in a Getinge autoclave (Getinge AB, Gothenburg, Sweden).

Potassium nitrate (KNO_3_) powder (Thermo Fisher Scientific, Waltham, MA) was mixed into each well until the target NO_3_-N concentration was established (within ±5% + 0.1 mg/L), measured with a TriOS NICO nitrate meter. The excess nitrate enriched stream water (intact and sterile) was saved in the cold room and used to replace the water removed for culturing. Once the nitrate levels were established, 30 of the wells were inoculated with ∼300 CFU each of a single enteropathogenic *E*. *coli* strain (*i.e.,* K99 or STEC O26), control wells were not inoculated. The *E*. *coli* levels and NO_3_-N concentrations of the water column in each microcosm were examined on 35 occasions, every 24 hrs for the first seven days then every 72 hrs until day 91. Colony counts above 500 CFU per 10 ml of water were too many to count, for this reason a 500 CFU/10 ml maximum was instituted. Nitrate concentrations were maintained by adding KNO_3_ as needed.

### Sample collection and bacterial culturing

All of the *E*. *coli* settled out of the water column in less than 24 hrs (as previously seen in the pilot experiment (Supplementary Information)), so all wells were briskly agitated with a sterile glass stirring spoon to resuspend the *E*. *coli* in the water column immediately prior to sample collection. Water column sample aliquots were diluted 1:10, 1:100, 1:1,000, 1:10,000, 1:100,000, and 1:1,000,000 with sterile MilliQ H_2_O to a final volume of 100 ml. Water samples of 10 µl were processed for each K99 and STEC O26 well. For negative control wells in sterile stream water 100 ml was sampled to ensure there was no contamination/growth. In the control wells containing intact stream water, background *E*. *coli* levels were monitored in the same method as the experimental wells with between 5–100 ml of water sampled, in increasing volumes until there were no *E*. *coli* grown. An additional 100 ml sample was processed from each control well 24 h after 0 CFU/100 ml was reached. All water samples less than 100 ml in size were diluted with sterile Milli-Q to a final volume of 100 ml. That 100 ml of water was then vacuum filtered through a single sterile 0.45 µm cellulose ester membrane filter (Merck KGaA, Darmstadt, Germany) and cultured.

Bacterial culturing followed United States Environmental Protection Agency method 1603 (EPA US, 2015). Each filter was placed onto a Difco Modified mTEC Agar (VWR, Radnor, PA, USA) plate, incubated at 37.5 °C for two hours, and then incubated at 45 °C for 18-20 h. Following incubation, colonies resembling *E. coli* (red/magenta colonies) were counted. Colony counts were calculated in CFU/10 ml and the amount removed from each vial or mesocosm for culture was replaced with an equal amount of the same water type containing the appropriate NO_3_-N concentration.

### Identification of *E*. *coli* present in the intact stream water microbiome

*Enterobacter cloacae,* commonly found in mammalian faeces is a bacterium that may produce the *β*-glucuronidase enzyme (Pearez, Berrocal & Berrocal, 1986). This enzyme is responsible for the red/magenta colony colour used to identify *E*. *coli* on Modified mTEC agar. It is also the gene typically targeted to identify *E*. *coli* using molecular methods. Therefore, they are easily mistaken for *E*. *coli* when using either of these methods. To ensure the identity of the colonies that were counted as *E*. *coli* were in fact *E*. *coli*, 96 colonies were randomly chosen from the background *E*. *coli* cultures across the 10–25 days for further characterisation. The selected colonies were purified on plate count agar (Merck KGaA, Darmstadt, Germany) and identified by matrix-assisted laser desorption ionization-time of flight (MALDI-TOF) mass spectrometry (Bruker, Billerica, CA, USA) using the “on slide formic acid extraction” method (Lévesque et al., 2015).

### Sample processing for molecular testing

To confirm the colonies grown from the inoculated intact river water microcosms were STEC O26 or K99, not imported background *E*. *coli*, 192 colonies (*e.g.*, 96 potential STEC O26 and 96 potential K99) phenotypically identified as *E*. *coli* by their red/magenta colour on Modified mTEC Agar were randomly selected from across the cultures, up to and including day 91. Additionally, to ensure no contamination of the control wells had occurred during sampling, 90 colonies (*e.g.*, 45 from STEC O26 intact control wells and 45 from K99 intact control wells) were randomly selected from across the intact control well cultures, up to and including day 25. Colonies were purified on Modified mTEC Agar. Genomic DNA was extracted from each purified colony using a boil preparation protocol; two or three colonies were suspended in 1 ml of Milli-Q H_2_O and heated at 100 °C for 10 min then centrifuged at 13,000 rpm for 5 min. The supernatant was aliquoted and used as DNA template.

### Molecular testing for target genes

We confirmed STEC O26 and K99 using a polymerase chain reaction (PCR) targeting *wzy* for O26 and the fimbril subunit for K99 (Table 1) (Roosendaal, Gaastra & De Graaf, 1984; Franck, Bosworth & Moon, 1998; Anklam et al., 2012). The detection limits of the STEC assays have been reported at 10^3^ CFU/ml (Anklam et al., 2012) with a specificity to sensitivity ratio at 92%:91% for O26 strains (Browne et al., 2018). The K99 primer has been reported to be highly specific and sensitive (Franck, Bosworth & Moon, 1998) however no exact limits were published. Using a positive control we determined that detection was best when there was at least 2 ng/µl of DNA template per reaction.

**Table 1:** Details of the oligonucleotide primers used in this study.

Gene	Primer sequences	Product size	Reference
*uidA*	Forward: 5′ AGTGTGATATCTACCCGCTT-3′ Reverse: 5′ AGAACGGTTTGTGGTTAATCAG-3′	84 bp	Anklam et al. (2012)
*wzy* O26	Forward: 5′AGCGTATGTTGATATATTTAATGTC-3′ Reverse: 5′AATGTGGTCCCAAGGAATAAA-3′	141 bp	Anklam et al. (2012)
*K99*	Forward: 5′ TATTATCTTAGGTGGTATGG-3′ Reverse: 5′ GGTATCCTTTAGCAGCAGTATTTC-3′	314 bp	Roosendaal, Gaastra & De Graaf (1984)

Amplification reactions were performed in 20 µl reaction volumes. STEC O26 reactions each contained 0. 5 × iQ PerfeCTa^®^ qPCR ToughMix™, ROX™ (QIAGEN, Düsseldorf, Germany), 1 pM of each primer, and 2.5 µl of DNA template. K99 reactions contained 0. 5 × iQ PerfeCTa^®^ qPCR ToughMix™, ROX™ (QIAGEN, Düsseldorf, Germany), 0.5 µM of each primer, and 3 µl of DNA template.

Thermocycling for both reactions was performed in a SensoQuest labcycler (Biomedizinische Elektronik, Göttingen, Germany) using standard cycling conditions as described in Anklam et al. (2012) and Roosendaal, Gaastra & De Graaf (1984).

Amplification products were visualised using RedSafe*™* (iNtRON Biotechnology, Daejeon, Korea) following electrophoresis in 2% Tris-acetate-ethylenediamine tetraacetic acid agarose gels.

### Data analysis

Statistical analyses were performed in R (R Core Team, 2017). Generalised linear models (GLM; Poisson response) were used to examine the response of K99, STEC O26, and background *E*. *coli* (in the control wells containing intact stream water), concentrations to treatment with nitrate, water type (sterile or containing in-stream microbiome) and duration of treatment. *Post hoc* Tukey’s honestly significant difference (HSD) tests were performed on an ANOVA using the *AICcmodavg* package (Mazerolle, 2017) to identify significant factors associated with background *E*. *coli* persistence. Plots were made in *ggplot2* (Wickham, 2016).

## Results

### Molecular testing and identification

All 96 potential STEC O26 colonies tested for *wzy* O26 contained the gene and all 96 K99 were positive for the fimbril subunit locus confirming their identities. The 90 background *E*. *coli* colonies were negative for *wzy* O26 and the K99 fimbril subunit but positive for *uidA*.

Additionally, all but three of the 96 colonies chosen from the background *E*. *coli* cultures were confirmed as *E*. *coli* by MALDI-TOF. The three that were not *E*. *coli* were identified as *Enterobacter cloacae*.

### Microcosm results

**Figure 1:**
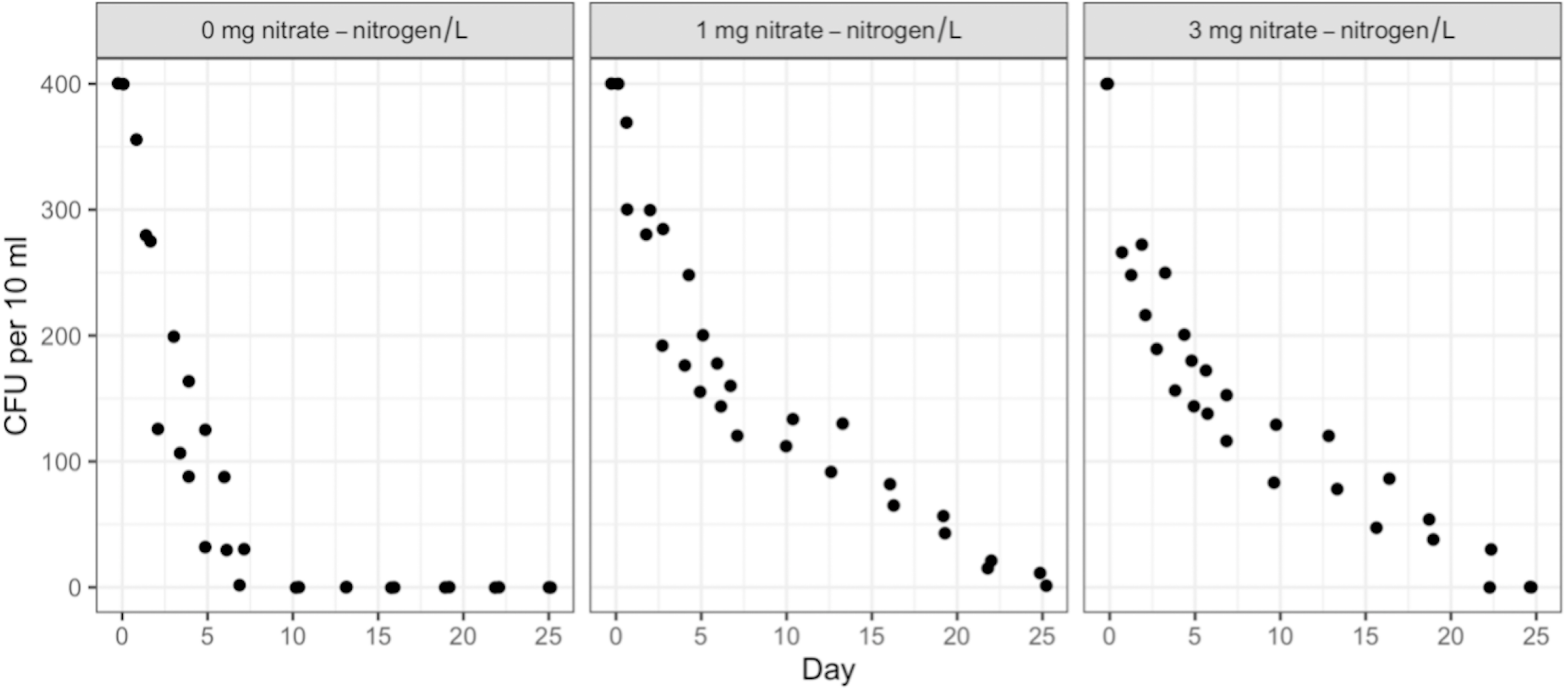
Averaged background *E*. *coli/E*. *cloacae* group die-off rates in the intact stream water at 0, 1, and 3 mg NO_3_-N/L concentrations.

**Figure 2:**
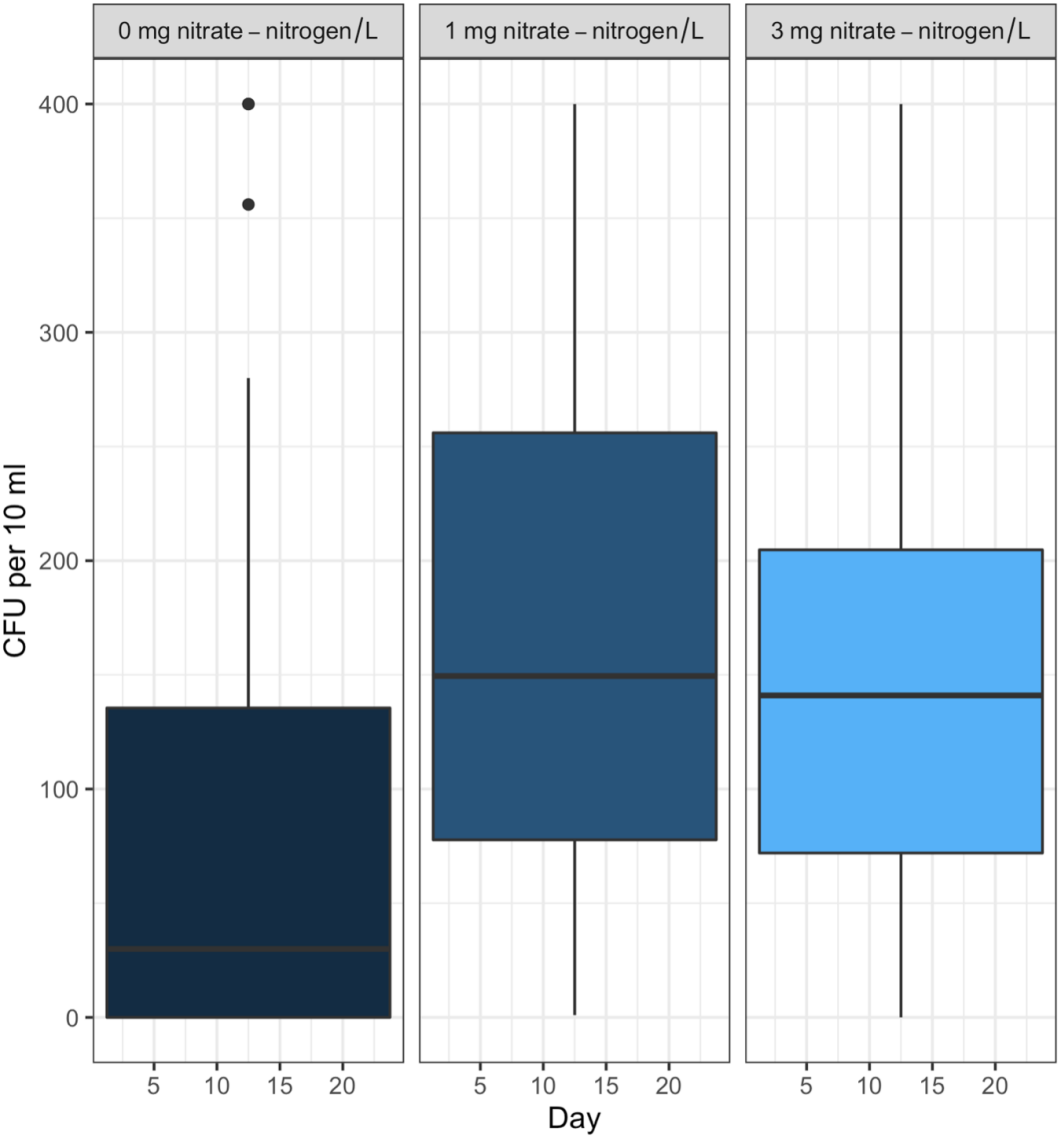
Mean number of background *E. coli/E*. *cloacae* group in the intact stream water at 0, 1, and 3 mg NO_3_-N/L concentrations.

Duration, NO_3_-N concentration, and water type (sterile or containing in-stream microbiota) had no measurable effect on STEC O26 or K99 growth or persistence (Table S2). Both strains attained 500 CFU/10 ml of water within 24 h of inoculation and maintained that level for the full 91 days. However, the survival of the background *E*. *coli*/*E*. *cloacae* group, imported as part of the in-stream microbiome in the intact stream water, increased non-linearly with NO_3_-N concentration and decreased with time (Figs. 1 & 2).

**Figure 3:**
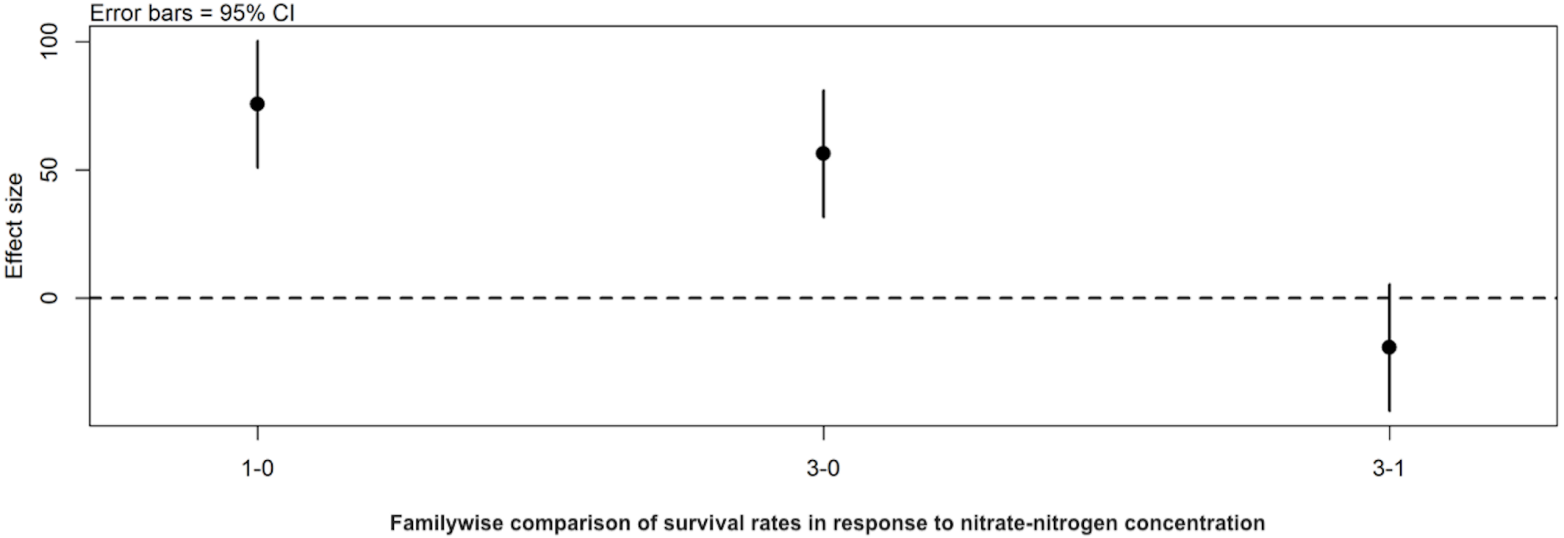
Familywise comparison of the effects NO_3_-N/L concentration had on the mean differences in background *E*. *coli*/*E*. *cloacae* group survival. Familywise comparison performed using a *post hoc* Tukey’s HSD test, of the effect of NO_3_-N concentration on the mean differences in background *E*. *coli*/*E*. *cloacae* group survival. Persistence in 3 mg NO_3_-N/L was similar to that in 1 mg NO_3_-N/L (Tukey’s HSD: *df* = 2, P adj. =0.153); but survival in 0 mg NO_3_-N/L was shorter than 1 mg/L (Tukey’s HSD: *df* = 2, P adj. =0.000) and 3 mg/L NO_3_-N (Tukey’s HSD: *df* = 2, P adj. =0.000).

*Post hoc* comparisons using the Tukey HSD test identified differences in background *E*. *coli* and *E*. *cloacae* group survival in NO_3_-N concentrations of 3 mg/L NO_3_-N and 1 mg/L NO_3_-N were similar (Tukey’s HSD: *df* = 2, P adj. = 0.153); but that survival in 0 mg/L NO_3_-N was significantly shorter than in 1 mg/L (Tukey’s HSD: *df* = 2, P adj. = 0.000) and 3 mg/L NO_3_-N (Tukey’s HSD: *df* = 2, P adj. =0.000) (Fig. 3, Table S2). The background *E*. *coli*/*E*. *cloacae* group were either no longer culturable or dead by day 10 in 0 mg/L NO_3_-N; but survived up to 15 days longer in 1 and 3 mg/L NO_3_-N with significant differences in survival rate a result of duration (Table S3).

## Discussion

This study is novel in that it investigated the effects of nitrate enrichment on animal and human enteropathogenic *E*. *coli* strains as well as in-stream sourced *E*. *coli* and *E*. *cloacae* group survival in the water column*.* It is also novel in its use of New Zealand sourced enteropathogenic *E*. *coli* strains in combination with New Zealand water column microbiota to determine whether in-stream microbiota do in fact remediate enteric/pathogenic bacterial pollution as has been hypothesised (Wanjugi & Harwood, 2013a; Ravva, Sarreal & Mandrell, 2014a). The effects of nutrient pollution and eutrophication on human and animal enteropathogenic *E*. *coli* strains in freshwater systems are poorly understood. One reason for this is that recreational water samples are rarely put through the additional testing necessary to identify specific enteropathogenic strains of *E*. *coli* as it can be time consuming and costly (Cooley et al., 2013; Tarr et al., 2019). However, enteropathogenic bacteria can be identified quickly and accurately using other techniques; molecular and MALDI-TOF (Anklam et al., 2012; Ragupathi et al., 2018).

That there was no measurable difference in enteropathogenic *E*. *coli* growth in response to increased nitrate concentrations was surprising; extending the length of the experiment and/or reducing the number of bacteria introduced should be investigated further. That K99 and STEC O26 were able to persist and grow in the microcosms regardless of NO_3_-N levels is an important finding supported by other studies where STECs, notably O157, have demonstrated extended persistence in aquatic environments (Korajkic et al., 2019b; Wang, Deering & Kim, 2020).

Interestingly, the background *E*. *coli*/*E*. *cloacae* group imported as part of the microbiome in the intact stream water did respond to NO_3_-N concentration. The response was not linear with increasing enrichment; treatments with 1 mg NO_3_-N/L having the highest retention and longest persistence time followed by 3 mg NO_3_-N/L and then 0 mg NO_3_-N/L. This may be due to death or the organisms no longer being culturable; viable but not culturable bacterial cells are understood to occur in laboratories (Ding et al., 2017; Liu et al., 2017). That said, further work on characterising the background *E*. *coli*/*E*. *cloacae* group to better understand their response to in-stream eutrophication is needed.

An unexpected finding was the speed at which the two enteropathogenic strains formed biofilms. Within 24 h, water column sampling without resuspension of the bacterial biofilms through mechanical agitation resulted in zero *E*. *coli* colonies grown. This is important from a monitoring perspective as recreational water monitoring only uses samples from the water column for microbial water quality and recreational safety assessments. Streams and rivers are mobile systems where mixing occurs regularly. However, many streams are slow moving or intermittent, and even those with high flows have slower flowing runs and pools. In areas where mixing is reduced, *E*. *coli* quickly fall out of the water column and adsorb to substrates, periphyton, and/or form biofilms on the water surface (Moreira et al., 2012; Vogeleer et al., 2014). For this reason, the water column may not be the best substrate to monitor in recreational waterways when attempting to assess the risk of enteropathogenic *E*. *coli* strains (Davis et al., 2021). In this experiment water flow and substrates such as rocks or sediment were not used, to ensure the manipulated variables were responsible for any observed differences. Future work should focus on the addition of different substrates, oxygenation levels, and water flow regimes and characterisation of the biofilms and their role in enteropathogenic *E. coli* survival.

Finally, and most importantly, in-stream microbiota had no measurable effect on the growth or persistence of either K99 or STEC O26. Other studies have suggested that survival of enteric/faecal bacteria, both commensal and pathogenic, may be mediated by aquatic microbes through competition and/or predation (Ravva, Sarreal & Mandrell, 2014b; Korajkic et al., 2019a). A study by Wanjugi & Harwood (2013a) and Wanjugi & Harwood (2013b) found that the presence of in-stream microbiota was the most important factor in remediating *E*. *coli* in the water column and sediments, but that STEC O157 displayed extended persistence. While their study used a different STEC serotype, took place over a much shorter time frame, at higher temperatures (5–10 °C higher than in our experiments), and included sediments, a similar resilience to in-stream microbiota and extended persistence was demonstrated by the enteropathogenic *E*. *coli* in our study (Wanjugi & Harwood, 2013b). Reductions in colony counts were observed within the first five days Wanjugi & Harwood’s (2013a) study. This did not happen in our study. There was no effect on either enteropathogenic strain that was attributable to in-stream microbiota. Our study used no substrate/sediment, only intact stream water. Considering the speed with which biofilm accumulation occurred, ongoing work is needed to determine whether the addition of benthic substrates could be important to the persistence/remediation of human and animal enteropathogenic *E*. *coli* strains in aquatic habitats. This next step may help identify an overlooked habitat for in-stream human and animal enteropathogenic *E*. *coli* strain sequestration or an environment hosting species capable of remediating enteropathogenic *E*. *coli* from aquatic systems.

While international studies on human pathogens have mixed findings on whether predation and competition in aquatic systems are important limiting factors to persistence or drivers of virulence (Erken, Lutz & McDougald, 2013; Mauro et al., 2013; Schmidt, Shringi & Besser, 2016), neither was found to be a significant factor for human and animal enteropathogenic *E*. *coli* strains in this experiment. In a realistic scenario, rivers or lakes reaching and maintaining NO_3_-N concentrations above 3 mg/L are likely to have other in-stream changes related to eutrophication and ecosystem distress, primarily, periphyton blooms or overgrowth of macrophytes (Camargo & Alonso, 2006). The ramifications of periphyton and macrophyte overgrowth (*e.g.*, fluctuating dissolved oxygen levels and changes in the type/amount of food available) may benefit human and animal pathogenic bacteria that are metabolically diverse, and are capable of surviving in both anoxic and hyperoxic environments. Conversely, bactivorous organisms may not be as adaptable to these conditions, therefore reducing predation (Blount, 2015).

## Conclusions

It is important we understand the potential impacts of rising nitrogen enrichment on our recreational and drinking water sources (Ward et al., 2018; Sprong et al., 2020). Nitrate-nitrogen concentrations at or exceeding those used in this study are being documented in waterways and aquifers, both nationally and globally (Canning, 2020; Tregurtha, 2020). The co-occurrence of elevated NO_3_-N concentrations and livestock faeces potentially carrying human and animal pathogens in freshwater systems is a direct result of catchment management and should have us questioning what effect excess nitrogen in our waterways is having on aquatic microbial communities and how that may affect human health (Dodds et al., 2009; Snelder, McDowell & Fraser, 2017). Therefore, aiming to reduce both nutrient and microbial pollution entering freshwater systems is the best way to protect all water for human and non-human life.

##  Supplemental Information

10.7717/peerj.13914/supp-1 Supplemental Information 1 Raw data

10.7717/peerj.13914/supp-2 Supplemental Information 2 STEC O26 and K99 raw data documenting CFU at each sample time across the 91 days of the experiment in the three NO_3_-N/L concentrations (*e.g.*, 0, 1, and 3) and both water types (0 - sterile stream water, 1 - intact stream water)

10.7717/peerj.13914/supp-3 Supplemental Information 3 Raw data of the background *E*. *coli* die off rates from the three intact stream water replicates at each nitrate-nitrogen concentration (*e.g.*, 0, 1, and 3 mg/l)

10.7717/peerj.13914/supp-4 Supplemental Information 4 The preliminary study referenced as supplementary information in the main article

10.7717/peerj.13914/supp-5 Supplemental Information 5 Rscript for generalised linear models used in main experiment

10.7717/peerj.13914/supp-6 Supplemental Information 6 Rscript used for ANOVA and Tukey’s HSD

## Additional Information and Declarations

### Data Availability

The following information was supplied regarding data availability: The raw data is available in the Supplemental Files.
