# Supplementary material for: Nitrate enrichment does not affect enteropathogenic Escherichia coli in aquatic microcosms but may affect other strains present in aquatic habitats
Source: PeerJ. 2022 Sep 27;10:e13914. doi: 10.7717/peerj.13914 (PMC9524367; doi:10.7717/peerj.13914)
Supplement: Supplemental Information 4 [file peerj-10-13914-s004.docx]

**Supplementary Information - Pilot experiment**

The primary aims of the pilot experiment were to: expand and refine the effects of a greater range of nitrate-nitrogen (NO_3_-N) concentrations; determine whether there was any response to cellular debris in the water; and to quantify any STEC strain specific responses.

**Methods**

**Bacteria**

For the pilot, two strains of Shiga-toxin producing *Escherichia* *coli* (STEC) O157 (NZRM 4274 (ESR - NZRM culture collection), and STEC O26 (NZRM 3537 (Browne et al., 2018; ESR - NZRM culture collection)) - were examined as model enteropathogenic bacteria and monitored for the number of colony forming units (CFU) present in the water column, to measure persistence, over 91 days.

**Water**

Two types of water were used in the pilot, Milli-Q and sterile stream water, unfiltered and containing cellular debris and other native chemicals. Stream water was collected from same stream and processed in the same manner as the sterile stream water in the primary experiment.

**Nitrate**

A gradient range, from one to 13 mg NO_3_-N/L in 0.5 NO_3_-N/L increments (i.e., 0, 0.5, 1.0, 1.5 mg NO_3_-N/L), was used for the pilot experiment.

**Experimental setup**

Once the 27 nitrate levels were established in 250 ml Schott bottles, 15 ml of the NO_3_-N enriched water was decanted into four, 20 ml bijou bottles as a negative control or for inoculation (e.g., four bottles of each water type – a negative control and three to be inoculated with one of the three bacterial strains - at each NO_3_-N concentration, n=162). The excess nitrate enriched water was saved in the cold room and used to replace the water removed for culturing. Due to the size of the bottles, testing NO_3_-N throughout the experiment was not possible. The pilot study was run consecutively over 18 months, not concurrently, due to space limitations.

Three bijou bottles at each of the 27 NO_3_-N concentrations were inoculated with ~100 CFU each of a single STEC strain (e.g., either O157 or O26). Negative control bottles were not inoculated. The bijou bottles with loosened tops were then held consecutively for 91 days in the dark at 10^o^C (+/- 0.5^o^C). Three replicates were performed for each treatment (n=486).

Sample collection and bacterial culturing

The STEC concentrations from each Bijou bottle were examined over the same time frame as the primary experiment. Similar to the primary experiment, both STECs formed biofilms in less than 24 hrs, so all bottles were briskly agitated to resuspend the STEC in the water column immediately prior to sample collection. Water samples ranged in volume from 2-500 µl, sample size was determined based on the previous culture results. For negative controls 2 ml was sampled to ensure there was no contamination/growth. All water samples were processed, cultured, and enumerated in the same manner as the primary experiment.

**Data analysis**

Statistical analyses were performed in R (R Core Team, 2013). An ANOVA (R Core Team, 2013) to ascertain whether colony forming units were affected by *E*. *coli* strain (e.g., STEC O157 or O26), NO_3_-N concentration, water type, and/or duration of treatment. A *Post hoc* Tukey honestly significant difference (HSD) test was performed using the *AICcmodavg* package (Mazerolle & Mazerolle, 2017) to identify significant factors. Plots were made using the ggplot2 package version 3.3.3 (Wickham, 2016).

**Results**

Neither strain (*P*=0975) nor water type (*P=*0.987) affected STEC growth or persistence; however, NO_3_-N concentration (*P*<0.001) and duration (*P*<0.001) strongly influenced survival (Table 1). All statistically significant variation in growth and persistence was limited to the first seven days (*P*<0.001) and was restricted to NO_3_-N concentrations between 6.5 mg NO_3_-N/L to 8.5 mg NO_3_-N/L (*P*<0.001). Growth in 12.0 and 12.5 mg NO_3_-N/L was noticeably slower in achieving 500 CFU/10 ml (taking ~48 hours (*P*<0.001)); but once it achieved 500 CFU/10 ml it remained there. All other NO_3_-N concentrations reached 500 CFU/10 ml by day two and remained there till the end of the experiment (i.e., day 91).

Table 1. Summary of the effect duration, water type, and NO_3_-N concentration had on STEC growth using ANOVA. Bold denotes a significance of 0.01 level or higher. NO_3_-N concentrations and days that significantly affected STEC growth determined using *post hoc* Tukey’s HSD tests are documented in the table.

| ANOVA |  | z value | P-value |
| --- | --- | --- | --- |
| K99 and O26 CFUs |  |  |  |
|  | Day | 5.386 | **<0.001** |
|  | NO_3_-N concentration | 56,577 | **<0.001** |
|  | Strain | 0.005 | 0.975 |
|  | Water type | 0.001 | 0.987 |
| Tukey’s HSD |  |  |  |
|  | 6.5-8.5 mg NO_3_-N/L |  | **<0.001** |
|  | 12-12.5 mg NO_3_-N/L |  | **<0.001** |
|  |  |  |  |
|  | Days 1-7:10-91 |  | **<0.001** |
